# Supplementary material for: Validation and Optimization of PURE Ribosome Display for Screening Synthetic Nanobody Libraries
Source: Antibodies (Basel). 2025 May 2;14(2):39. doi: 10.3390/antib14020039 (PMC12101283; doi:10.3390/antib14020039)
Supplement: Supplementary file 1 [file antibodies-14-00039-s001.zip › antibodies-3561778-supplementary.pdf]

Supplementary Material

Validation and Optimization of PURE Ribosome Display for Screening Synthetic Nanobody Libraries

Bingying Liu and Daiwen Yang*

Department of Biological Sciences, National University of Singapore, 14 Science Drive 4, Singapore 117543

Table S1. Selection conditions for each round of biopanning to select EGFP-specific nanobodies using biotin elution method.

	Solid phase	EGFP-$(GGGGS)_4$-SBP volumn and concentration	Washing condition	Elution conditon
Round 1	10 µl Streptavidin magnetic beads resin(NEB)	100 µl, 200 nM	500 µl WTB, 3 times	50 µl 40 µM Biotin, 2 times
Round 2	Pierce™ Streptavidin coated plates (1 well)	100 µl, 100 nM	300 µl WTB, 5 times	100 µl 40 µM Biotin
Round 3	6 µl Streptavidin magnetic beads resin (NEB)	100 µl, 50 nM	500 µl WTB, 3 times	50 µl 40 µM Biotin, 2 times
Round 4	Pierce™ Streptavidin coated plates (1 well)	100 µl, 20 nM	300 µl WTB-T (0.1%), 5 times	100 µl 40 µM Biotin

Table S2. Selection conditions for every round of biopanning to select hFABP4-specific nanobodies after optimization. Pre-incubation with streptavidin-coated surface step was added at round 2 and round 4. Ni-NTA agarose resin was used in round 3 to reduce the streptavidin-specific binders' enrichment.

	Solid phase	Protein	Protein volumn and concentration	Pre-selection step	Washing condition	Elution conditon
Round 1	4 µl Dynabeads™ MyOne™ Streptavidin T1 resin	FABP4-$(GGGGS)_4$-SBP	100 µl, 200 nM	NA	500 µ WTB-T (0.1%), 3 times	50 µl 40 µM Biotin, 2 times
Round 2	Pierce™ Streptavidin coated plates (1 well)	FABP4-$(GGGGS)_4$-SBP	100 µl, 140 nM	Incubate in a streptavidin coated well for 30 min	300 µl WTB-T (0.1%), 5 times	100 µl 40 µM Biotin
Round 3	5 µl Ni-NTA agarose resin (Qiagen)	His-SUMO-FABP4	100 µl, 100 nM	NA	500 µ WTB-T (0.1%) containing 5 mM Imidazole, 5 times	50 µl 200 mM imidazole, 2 times
Round 4	Pierce™ Streptavidin coated plates (1 well)	FABP4-$(GGGGS)_4$-SBP	100 µl, 50 nM	Incubate in a streptavidin coated well for 30 min	300 µl WTB-T (0.2%), 5 times	100 µl 40 µM Biotin

Table S3. List of primers.

Primer name	Primer sequence (5' ---> 3')	Restriction site
RT_universal_Fr	ATGC**GGATCC**CAGGTTCAGCTG	BamHI
RT_universal_Rv	GACAGGAGGTTG**AAGCTT**	HindIII
FR1_Fr	GTTGAAAGCGGCGGCGGCCTGGTTCAGGCGGGCGGCAGCCTGCGTCTGAGCTGCGCGGCG	
CDR1_Rv	CCCGGCGCCTGACGGTACCA /TriMix * 7/GCTCGCCGCGCAGCTCAGAC	
FR2_Fr	TGGTACCGTCAGGCGCCGGGCAAAGAACGTGAATTCGTTGCG	
CDR2_Rv	CCTTTAACGCTATCCGCGTA/TriMix * 6/CGCAACGAATTCACGTTCTT	
CDR3_Fr	GCGGTTTACTACTGCGCGGCG/TriMix * 10/TACTGGGGCCAGGGCACCCAGGTTACCGTT	
FR4_Rv	GATTTT**AAGCTT**GCTGCTAACGGTAACCTGGGTGCCCT	HindIII
FR3 Aplf_Fr	TACGCGGATAGCGTTAAAGG	
FR3(AVYYCA)_Rv	CGCGCAGTAGTAAACCGC	
FR1 start_Fr	CAGCTGGTTGAAAGCGGCGGCGG	
FR1-2 Aplf_Rv	ACGGCCTTTAACGCTATCCGCGTA	
BamHI_FR1 start_Fr	ATGC**GGATCC**CAGGTTCAGCTGGTTGAAAGCGGCGG	BamHI
FR4_Rv_54oC	GATTTT**AAGCTT**GCTGCTAACG	HindIII
T7_transcripte_Fr	AGAGCAGATTGTACTGAGAGTGCACCA	
T7_transcripte_Rv	CCGCACACCAGTAAGGTGTG	

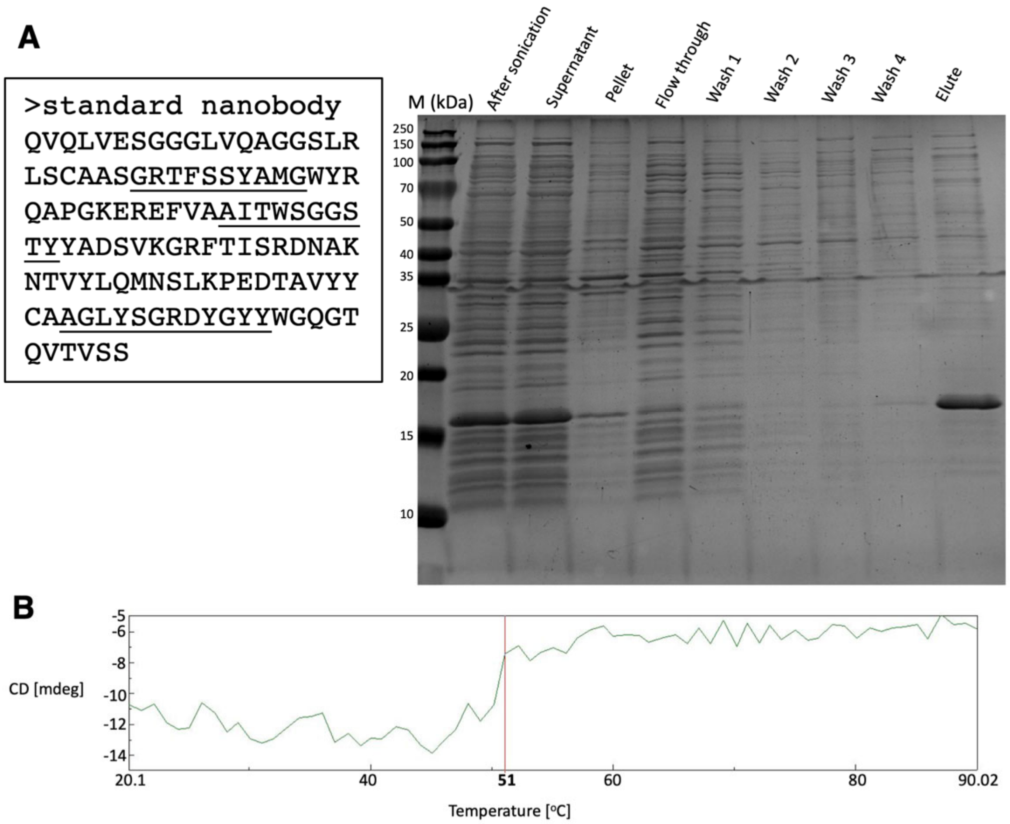

Figure S1. Nanobody framework design and universality analysis. *(A). Standard nanobody's sequence, and SDS-PAGE image about the purification process. Wash 1: 0 mM imidazole; Wash 2: 5 mM imidazole; Wash 3: 10 mM imidazole; Wash 4: 20 mM imidazole; Elute: 200 mM imidazole. (B). Change of ellipticity of the standard nanobody with temperature as monitored by CD.*

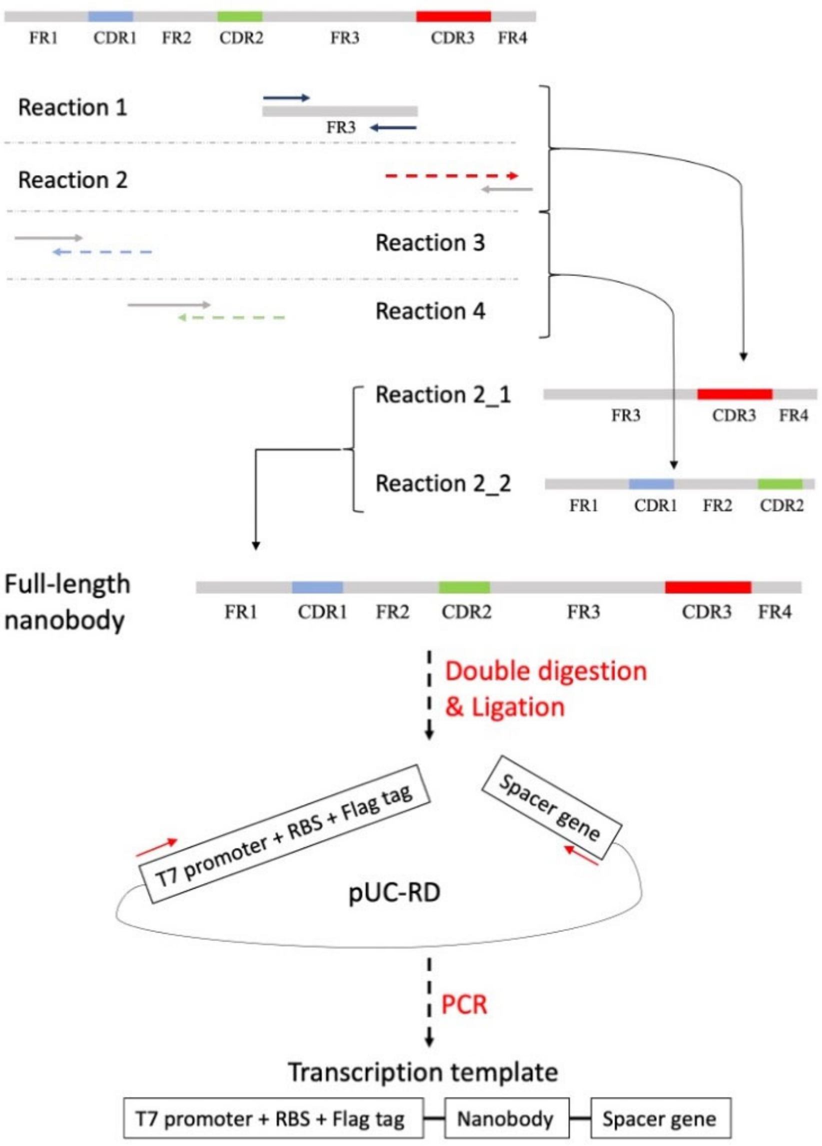

Figure S2. Flow diagram of synthetic nanobody library construction.

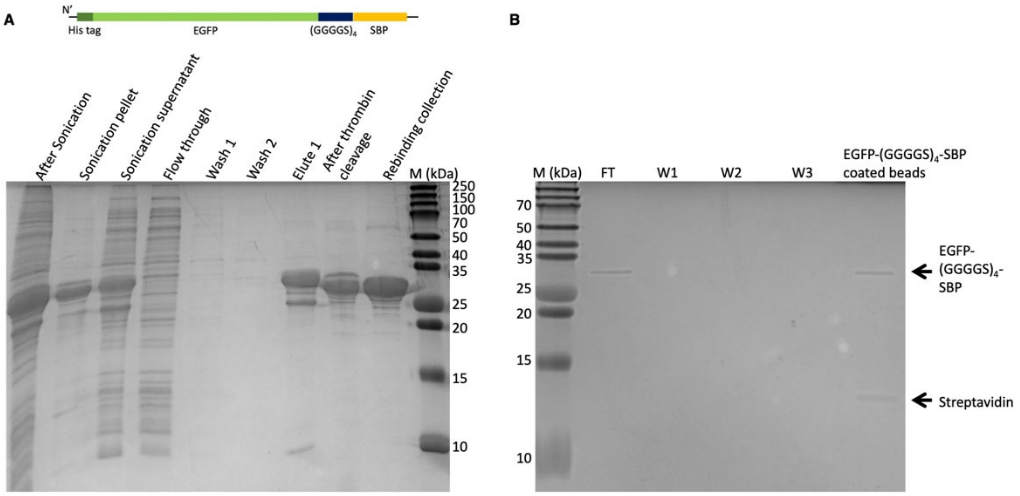

Figure S3. SDS-PAGE images of the protein purification and immobilization processes. *(A) His-EGFP-(GGGGS)$_4$-SBP construct diagram and purification process of His-EGFP-(GGGGS)$_4$-SBP. Wash 1: 0 mM imidazole; Wash 2: 5 mM imidazole; Wash 3: 10 mM imidazole; Wash 4: 20 mM imidazole; Elute 1: 200 mM imidazole. (B) SDS-PAGE image of EGFP-(GGGGS)$_4$-SBP interacting with streptavidin coated magnetic beads. FT: flow through after incubation. W1, W2, W3: three washes with 100 µl of PBS each time.*

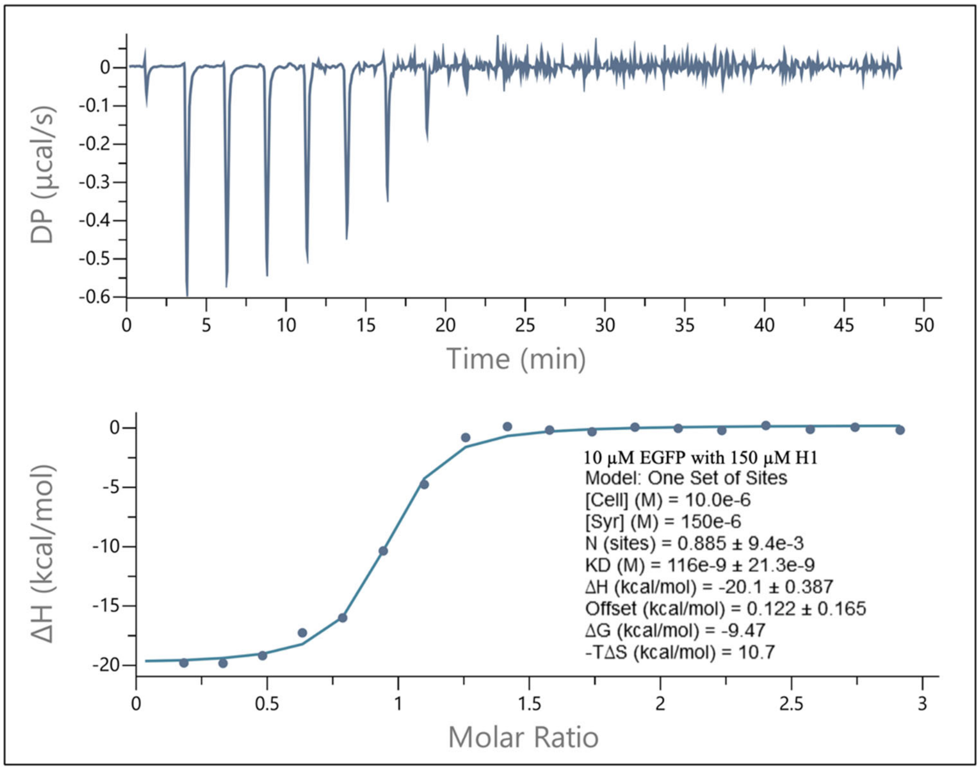

Figure S4. Analysis of the affinity of H1 with EGFP by ITC. Raw ITC data (upper) and fitted curves (lower) are shown. *10 μM EGFP was in ITC cell, while 150 μM H1 was in ITC syringe.*
